# Animal tracking moves community ecology: Opportunities and challenges

**DOI:** 10.1111/1365-2656.13698

**Published:** 2022-04-18

**Authors:** Raul Costa‐Pereira, Remington J. Moll, Brett R. Jesmer, Walter Jetz

**Affiliations:** ^1^ Departamento de Biologia Animal, Instituto de Biociências Universidade Estadual de Campinas Campinas Brazil; ^2^ Department of Natural Resources and the Environment University of New Hampshire Durham NH USA; ^3^ Department of Fish and Wildlife Conservation Virginia Tech Blacksburg VA USA; ^4^ Department of Ecology and Evolutionary Biology Yale University New Haven CT USA; ^5^ Center for Biodiversity and Global Change Yale University New Haven CT USA

**Keywords:** dispersal, ecological interactions, environmental niche, GPS‐tracking, intraspecific variation, remote sensing

## Abstract

1. Individual decisions regarding how, why and when organisms interact with one another and with their environment scale up to shape patterns and processes in communities. Recent evidence has firmly established the prevalence of intraspecific variation in nature and its relevance in community ecology, yet challenges associated with collecting data on large numbers of individual conspecifics and heterospecifics have hampered integration of individual variation into community ecology.
2. Nevertheless, recent technological and statistical advances in GPS‐tracking, remote sensing and behavioural ecology offer a toolbox for integrating intraspecific variation into community processes. More than simply describing where organisms go, movement data provide unique information about interactions and environmental associations from which a true individual‐to‐community framework can be built.
3. By linking the movement paths of both conspecifics and heterospecifics with environmental data, ecologists can now simultaneously quantify intraspecific and interspecific variation regarding the Eltonian (biotic interactions) and Grinnellian (environmental conditions) factors underpinning community assemblage and dynamics, yet substantial logistical and analytical challenges must be addressed for these approaches to realize their full potential.
4. Across communities, empirical integration of Eltonian and Grinnellian factors can support conservation applications and reveal metacommunity dynamics via tracking‐based dispersal data. As the logistical and analytical challenges associated with multi‐species tracking are surmounted, we envision a future where individual movements and their ecological and environmental signatures will bring resolution to many enduring issues in community ecology.

## Graphical abstract

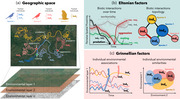

## Resumo

1. Decisões tomadas por indivíduos sobre como, por que e quando organismos interagem uns com ou outros e com o ambiente moldam padrões e processos em comunidades. Evidências recentes estabeleceram fortemente a prevalência da variação intraespecífica na natureza e sua relevância para a ecologia de comunidades. Entretanto, uma série de desafios associados com a coleta de dados empíricos em um número representativo de indivíduos conspecíficos e heterospecíficos tem dificultado a integração da variação individual na ecologia de comunidades.
2. No entanto, desenvolvimentos tecnológicos e estatísticos recentes em técnicas de rastreamento por GPS, sensoriamento remoto e ecologia comportamental oferecem as ferramentas necessárias para integrar a variação intraespecífica em processos na escala de comunidades. Mais do que simplesmente descrever onde diferentes organismos vão, dados de movimento animal fornecem informações únicas sobre interações bióticas e associações ambientais, a partir das quais um verdadeiro *framework* de indivíduos para comunidades pode ser construído.
3. Ao conectar trajetórias de movimento de conspecíficos e heterospecíficos com dados ambientais, ecólogos agora podem simultaneamente quantificar variações intra‐ e interespecíficas de fatores Eltonianos (interações bióticas) e Grinnellianos (condições ambientais) relacionados com a montagem de comunidades e suas dinâmicas. Entretanto, desafios logísticos e analíticos devem ser resolvidos antes que tal abordagem finalmente atinja seu potencial máximo.
4. Entre comunidades, a integração empírica de fatores Eltonianos e Grinnellianos pode fomentar aplicações para a conservação e revelar dinâmicas de metacomunidades via dados de dispersão baseados em biorastreamento. Conforme os desafios logísticos e analíticos associados com o biorastreamento simultâneo de indivíduos de múltiplas espécies sejam superados, nós antevemos um futuro no qual os movimentos de indivíduos e suas assinaturas ecológicas e ambientais trarão novas perspectivas para muitos dos problemas duradouros em ecologia de comunidades.

## 1 UNPACKING COMMUNITY PROCESSES

Ecologists have long sought to understand the forces governing the structure and functioning of the multilayered fabric of life, which involves recognizing a hierarchy of processes operating from individuals to communities (Levin, 1992). The inherent complexity of ecological systems has forced ecologists to balance realism and tractability in their models, which has resulted in a focus on species rather than individuals. Indeed, this widespread tendency is well justified given that interspecific differences are generally more conspicuous than intraspecific differences (Coulson, 2020). Nonetheless, the dynamics of animal communities are ultimately the products of individual decisions regarding how, why and when organisms move and interact with one another and with their environment (Potts et al., 2014; Schlägel et al., 2020; Spiegel et al., 2017). For instance, interspecific niche partitioning, a long‐recognized condition favouring species coexistence (MacArthur & Levins, 1967), emerges from variation in resource use within and between species (Costa‐Pereira, Araújo, et al., 2019). Both intraspecific and interspecific dietary niche differences are shaped by how conspecifics and heterospecifics navigate the trophic landscapes (e.g. by selecting food patches) (Pansu et al., 2019).

Although species‐level studies have greatly advanced our understanding about community assembly and functioning over the last half century, a rapidly growing body of literature has revealed the significance of intraspecific ecological variation in communities (Bolnick et al., 2011). Generalist populations are often composed of groups of individuals that are resource or habitat specialists (Ingram et al., 2018; Schirmer et al., 2020) and this intraspecific variation can have stronger ecological effects than interspecific variation (Des Roches et al., 2018; Harrison et al., 2019). Therefore, individual ecological diversity plays a key role in shaping dynamics at the level of populations, communities and ecosystems (Allgeier et al., 2020; Bolnick et al., 2011; Ingram et al., 2018; Schirmer et al., 2020). Individuals within communities differ in their traits (Violle et al., 2012), trophic niches (Costa‐Pereira, Araújo, et al., 2019), behaviours (Dantzer & Rubenstein, 2017; Harrison et al., 2019) and environmental associations (Carlson et al., 2021). Despite the importance of intraspecific and interspecific interactions and environmental associations in structuring communities (Thompson et al., 2020), understanding how these individual‐level processes scale up to shape community dynamics remains an outstanding gap in knowledge.

The empirical development of an individual‐to‐community approach has been hindered by two major challenges. First, because of the enormous logistical challenge of collecting data at an individual level at high spatiotemporal resolutions, there have been historical empirical limitations of incorporating individual variation in a multi‐species framework (Coulson, 2020). Second, studies have often focused on either interactions between organisms or environmental associations rather than both simultaneously (Soberón, 2007), creating conceptual and logistical discontinuities. We contend that movement data collected within a holistic conceptual framework and integrated across individuals and species has the potential to bring resolution to many enduring issues in community ecology.

## 2 LINKING INDIVIDUAL MOVEMENTS TO COMMUNITY PROCESSES

Recent advances in GPS‐tracking, biologging technologies (e.g. animal‐borne video, proximity loggers, PIT‐tags) and fine‐scale remotely sensed data now enable simultaneous quantification of how individuals interact with conspecifics, heterospecifics and their environment (Nathan et al., 2022; Tuia et al., 2022). These technological advances have facilitated tracking greater numbers of individuals across multiple co‐occurring species (e.g. Davidson et al., 2020; Johnson et al., 2013; Raymond et al., 2015) in both terrestrial and aquatic systems. The high spatiotemporal resolution of these data facilitates detailed quantification of interactions over long time‐scales—even lifetimes (Nathan et al., 2022). These ‘quantitative biographies’ have already yielded numerous new insights into the social behaviour of animals (King et al., 2018; Strandburg‐Peshkin et al., 2015; Strandburg‐Peshkin et al., 2017). However, these recent developments have rarely been applied in community‐level contexts (Bro‐Jørgensen et al., 2019) (see Section 9).

**FIGURE 1:**
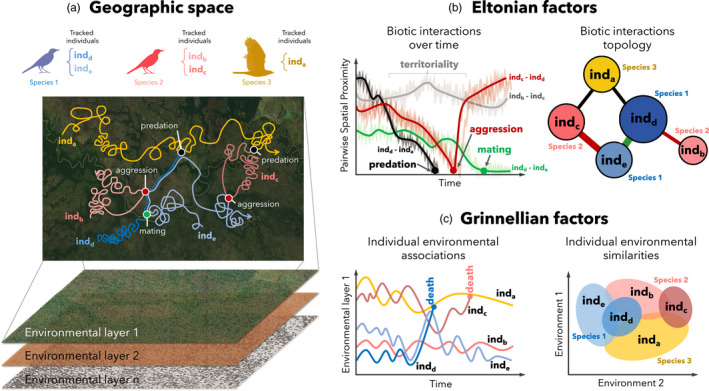
Eltonian and Grinnelian dynamics inferred from multi‐species tracking data. *Panel (a)*: Tracks of five individuals from three different species reveal intraspecific and interspecific interactions through time, thereby enabling the construction of interaction topologies including both conspecifics and heterospecifics at an individual level. *Panel (b)*: Interactions in the Eltonian arena can be mapped via temporally explicit tracks, allowing for spatiotemporal analysis of interactions across landscapes. Therefore, the intersection of tracks with environmental data (e.g. remote sensing layers) in space and time quantifies environmental associations and facilitates assessments of population‐ and community‐wide Grinnellian niche partitioning

Tracking of individuals in a multi‐species framework (Figure 1) has the potential to reveal key processes underpinning communities (Bro‐Jørgensen et al., 2019; Milles et al., 2020; Schlägel et al., 2020). First, movement data facilitate more than just mapping biotic interactions at fine spatiotemporal scales. When coupled with high‐resolution environmental data, individual movements also describe fine‐scale variation in environmental associations both within and between species, providing fine‐scale data to investigate how environmental filters drive community patterns (Bastille‐Rousseau & Wittemyer, 2019). At broader scales, movement data can help elucidate metacommunity and regional diversity dynamics by providing a window through which we can observe dispersal and migration and their consequences on both source and receiver communities (Bauer & Hoye, 2014; Jesmer et al., 2018; Schlägel et al., 2020).

## 3 USING MOVEMENT DATA TO INTEGRATE ELTONIAN AND GRINNELLIAN COMMUNITY PROCESSES

Interactions between conspecifics, heterospecifics and the environment form the backbone of community ecology. Understanding general patterns of biodiversity should therefore involve uncovering both the impacts and responses of an organism in relation to other organisms (i.e. biotic interactions)—referred to as Eltonian factors (Chase & Leibold, 2003; Soberón, 2007)—and the environmental conditions necessary to sustain neutral or positive fitness values (i.e. environmental associations)—referred to as Grinnellian factors (Thompson et al., 2020). Although ongoing work seeks to integrate these concepts under a unified theory of ecological niches (Chase & Leibold, 2003; Gravel et al., 2019; Peterson et al., 2011; Potts et al., 2014), Eltonian and Grinnellian factors have traditionally been studied in isolation. This is due to a paucity of data capable of supporting such an integration (i.e. data for interacting organisms collected over consistent spatial and temporal scales) (Soberón, 2007). By linking modern remote‐sensing data with auxiliary biologging technologies (e.g. proximity loggers, animal‐borne video), the tracking of individual organisms in a multi‐species framework may now overcome these historical challenges and support synthesis. Here we offer a framework for leveraging these new technology‐driven opportunities in community ecology and explore the key challenges that must be overcome to make this vision a reality.

## 4 ELTONIAN FACTORS CAPTURED WITH TRACKING DATA

Elton defined the niche as the role a species plays in a given community, particularly ‘*its relations to food and enemies*’ (MacArthur & Levins, 1967). Contemporary theory has operationalized this concept by focusing on the impacts one species has on other organisms (Chase & Leibold, 2003; Letten et al., 2017). The influence of biotic interactions that shape this niche space, such as predation and competition, can be inferred probabilistically from analysis of individual movement data which is both spatially and temporally explicit (Isbell et al., 2018; Milner et al., 2021; Nathan et al., 2022; Schlägel et al., 2019; Suraci et al., 2022; Villegas‐Ríos et al., 2020). These Eltonian factors vary by magnitude, frequency and type, thereby encompassing interactions often described at the species level in classical theory (Wootton, 1994). Importantly, movement data facilitate insight both into intraspecific (King et al., 2018; Strandburg‐Peshkin et al., 2015) and interspecific biotic interactions (Montiglio et al., 2019), thereby enabling the construction of community topologies (e.g. food webs) using individuals rather than species as functional units (Figure 1).

Classical work on Eltonian factors for species pairs is built upon consumer‐resource models (Murdoch et al., 2003) and has since been expanded to include non‐consumptive factors, whereby phenotypic changes mitigate negative interactions (e.g. anti‐predator behaviours; Abrams, 1995). Sequential and simultaneous location data from multiple moving organisms (both conspecifics and heterospecifics) enable inference regarding Eltonian factors from patterns of attraction, repulsion or neutrality (Milner et al., 2021; Potts et al., 2014; Schlägel et al., 2019). For example, direct predation is inferred via cessation of motion or clustered locations surrounding kill sites (Anderson & Lindzey, 2003). When combined with auxiliary data (e.g. diet composition) and other biologging measures (e.g. accelerometers, heart rate loggers) (Williams et al., 2020), individual movements can reveal how consumptive and non‐consumptive factors combine to shape fitness‐optimizing behaviour. For instance, a proximate threat of predator encounter can cause prey to move to less nutritious, but safer, foraging patches (Barnier et al., 2014) and lead to trophic cascades (Ford et al., 2014). Certain Eltonian factors are linked to symmetric spatiotemporal associations (e.g. positive–positive as in cooperative foraging), while others result in asymmetries (e.g. negative–positive, as in prey fleeing a cursorial predator) (Villegas‐Ríos et al., 2020). Such asymmetries underscore the need for careful analyses to disentangle pattern from process (Freilich et al., 2018).

Movement data can also reveal indirect Eltonian factors, which occur when the effects of one species on another are mediated by a third species (e.g. apparent competition). Like their direct counterparts, indirect factors propagate along consumptive and non‐consumptive pathways (Abrams, 1995; Gil et al., 2018). Importantly, these factors have analogues on the individual organizational level, although studies at this resolution remain rare. For example, multi‐species tracking studies on carnivores and ungulates have revealed complex intraguild interspecific avoidance based upon dominance hierarchies and associated mechanisms of community niche partitioning (Dröge et al., 2017; Vanak et al., 2013). However, little understanding of how individual variation influences such community dynamics exists (Gil et al., 2018). Multi‐species movement studies represent an exciting new research avenue because the mechanistic underpinnings of complex community processes have likely been masked by aggregative spatiotemporal patterns at the population level (Bolnick et al., 2011; Peterson et al., 2020; Potts et al., 2014).

## 5 GRINNELLIAN FACTORS CAPTURED WITH TRACKING DATA

Since Grinnell's original conceptualization, a species' environmental niche has been defined as the suite of conditions necessary to sustain viable populations (Chase & Leibold, 2003). Ecologists' ability to quantify such Grinnellian factors is rapidly developing as advances in sensing technologies, data processing and computational modelling have led to a growing number of remotely sensed products that capture environmental conditions at increasingly fine spectral, temporal and spatial resolution at a near‐global extent (Anderson, 2018; He et al., 2015; Jetz et al., 2016; Mertes et al., 2020; Randin et al., 2020; Tuia et al., 2022; Wilson & Jetz, 2016). Furthermore, airborne sensing (e.g. LiDAR) offers even higher spectral and spatiotemporal resolution and detailed habitat characterizations (Asner et al., 2017; Carrasco et al., 2019). Simultaneously, tracking technologies have become more miniaturized and efficient, thereby facilitating tracking of smaller‐bodied species and finer‐scale temporal sampling of animal movement (Kays et al., 2015; Wikelski et al., 2007). Intersecting multi‐species movement tracks with remotely sensed data allows quantifying Grinnellian factors for conspecifics and heterospecifics in *n*‐dimensional environmental niche space (Carlson et al., 2021) (Figure 1). In turn, this niche space can be projected into geographical space to map distributional areas (Colwell & Rangel, 2009), allowing ecologists to assess whether and how intraspecific and interspecific variation in environmental niche space affects community dynamics (Figure 1).

Fundamental to quantifying Grinnellian factors is an understanding of the fitness consequences of environmental conditions (Pulliam, 2000). Although the many data types (e.g. presence only, presence–absence survey data, expert range maps) used to determine Grinnellian niches require ancillary studies to quantify individual fitness, tracking data can simultaneously assess fitness and the environmental conditions experienced by individuals from multiple co‐occurring species. For example, individual movement patterns enable ecologists to remotely evaluate survival, parturition and recruitment across heterogeneous landscape conditions (DeMars et al., 2013; Hooven et al., 2022). In this way, spatiotemporally explicit movement data permit identifying when and where individuals, populations and species experience favourable conditions.

## 6 TOWARDS AN INTEGRATED UNDERSTANDING

Movement data enable joint assessment, and thus integration, of Eltonian and Grinnellian factors at the level of individuals, populations and communities (Figure 1). This integration allows ecologists to unify niche concepts and may illuminate how intraspecific niche variation influences classical paradigms in community ecology (e.g. species coexistence) (Bolnick et al., 2011; Milles et al., 2020). To successfully integrate across niche concepts and organismal scales, movement models need to jointly estimate (a) the impacts individuals have on each other (both conspecifics and heterospecifics) and (b) the conditions individuals need to sustain positive fitness values. Such joint quantifications are enabled by a growing set of methods and tools, particularly those from social behavioural research (Bro‐Jørgensen et al., 2019; King et al., 2018; Nathan et al., 2022; Strandburg‐Peshkin et al., 2015; Tuia et al., 2022), but will require judgement around balancing analytical complexity with logistical and interpretational tractability (see Section 9).

The integrative potential of movement data for community ecology is further enhanced by complementary sources of ecological data. For instance, when combined with movement data, a diverse array of methods (e.g. stable isotopes, DNA metabarcoding, video and proximity‐enabled collars) now support the characterization of intraspecific and interspecific niche partitioning and plasticity. Such auxiliary information enables deeper insights into the behavioural mechanisms shaping niches (Bastille‐Rousseau & Wittemyer, 2019). For instance, video and proximity‐enabled tracking devices supply fine‐scale behavioural data on interspecific interactions that may not be fully captured by locational data alone (e.g. physical contact between individuals) (Lavelle et al., 2012; Moll et al., 2007). Likewise, combining animal tracking with stable isotope or DNA metabarcoding data can help uncover how foraging decisions may influence patterns of movement and space use (Atkins et al., 2019; Bradshaw et al., 2017; Votier et al., 2010). To date, studies of such sophistication have predominantly focused on individuals of a single species (see Section 9), but multi‐species extensions represent an exciting avenue for future research.

## 7 FROM SINGLE TO MULTIPLE COMMUNITIES AND ECOSYSTEMS

**FIGURE 2:**
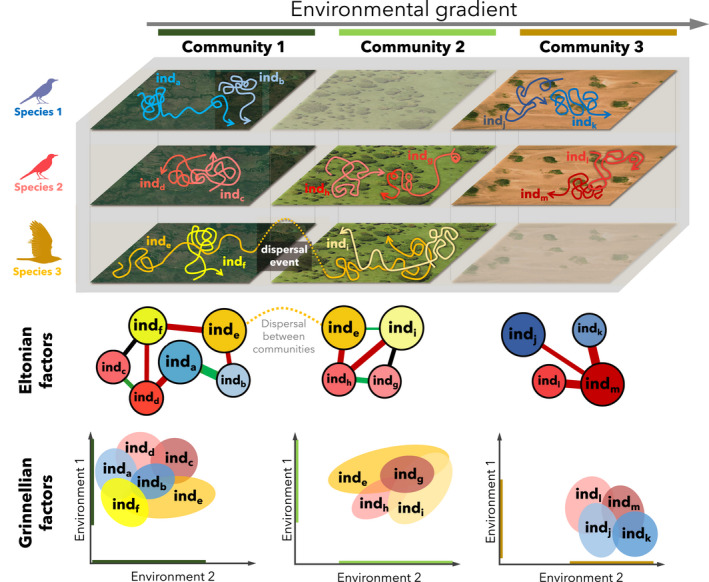
Inferring Eltonian and Grinnelian dynamics from single to multiple communities. Panels depict how communities vary by the number of species present, the number of individuals per species, the movement behaviour of such individuals and local environmental characteristics. Community 1 harbours three tracked species, while in community 2, species 2 experiences competition release and in community 3 species 1 and 2 experience predation release. The dispersal event (individual_e_) depicted between communities 1 and 2 emphasizes the power of tracking data to quantify biotic links across communities

The ability of movement data to quantify processes shaping community dynamics extends beyond single communities. As Eltonian and Grinnellian factors vary along environmental gradients (Gravel et al., 2019), tracking individuals from sets of species across multiple communities will enlighten how local contexts (e.g. local diversity, environmental heterogeneity) affect individual interactions and thus species niches (Figure 2). For instance, comparing how interactions between conspecifics and heterospecifics change along temperature gradients or with the presence of predators are, respectively, promising research agendas for understanding how climate change and defaunation impact the organization of communities (Barnier et al., 2014; Veldhuis et al., 2020).

Community ecology has experienced a change in its focus from single to multiple, connected communities. Metacommunity theory proposes that the interplay of within‐ and among‐community factors drives regional biodiversity patterns (Leibold & Chase, 2017; Thompson et al., 2020). In this context, individual movements can provide critical information about among‐community connectivity via biotic links. Dispersal is the key process governing spatial dynamics across metacommunities (Leibold & Chase, 2017; Thompson et al., 2020); therefore, tracking data can answer open questions about the causes of individual dispersal and their implications for populations and communities (Schlägel et al., 2020). For instance, mapping pre‐dispersal interactions and environmental associations may reveal factors triggering dispersal by specific individuals within populations, as well as how dispersal impacts both recipient and source metacommunities (Figure 2). Finally, individual movements can also help understand links among ecosystems, sometimes separated by thousands of kilometres (Alerstam et al., 2003; Bauer & Hoye, 2014). Because animals move substantial amounts of matter and energy across ecosystem boundaries (Schmitz et al., 2018), movement data can uncover the magnitude of spatial coupling between ecosystems, allowing the development of models capable of predicting the impacts of disruptions in meta‐ecosystem fluxes due to anthropogenic barriers to movement.

## 8 FROM BASIC TO APPLIED COMMUNITY ECOLOGY

In a rapidly changing world, the combination of animal movement and remote sensing data offers powerful conservation perspectives for safeguarding biodiversity (Nathan et al., 2022; Tuia et al., 2022). For example, Grinnellian niche assessments support myriad uses for modelling fine‐scale species distributions and animal–habitat relationships. Such assessments are especially effective for herbivores because remote sensing data can often directly capture their relevant niche factors (e.g. NDVI), although matching remote sensing product resolution with fine‐scale tracking data remains an ongoing challenge, especially for predators (Suraci et al., 2022). The output of such models can help predict the current and future composition of communities, which, in turn, can strategically guide conservation action (e.g. identify diversity hotspots) (Hazen et al., 2013). For instance, by tracking individuals from several (*n* = 6–21) species of birds and mammals inhabiting the same region at the same time, specific marine regions critical for breeding and foraging at the community level were identified (Davies et al., 2021; Raymond et al., 2015). These emerging patterns from individual‐level data in a multi‐species context are vital for informing policy regarding the establishment of protected areas.

Individual movement data also have the potential to support mechanistic and spatiotemporally explicit predictions of how anthropogenic impacts (e.g. land use change, species introductions) will change interactions between conspecifics and heterospecifics (Kays et al., 2015; Nathan et al., 2022; Veldhuis et al., 2020). For example, while it is widely recognized that wildlife often responds to human activity by becoming more nocturnal or limiting movements (Gaynor et al., 2018; Tucker et al., 2018), individual‐level analyses could reveal how responses to human activity vary within and across species. In turn, individual behavioural responses are likely to alter the dynamics of populations and communities (Bolnick et al., 2011; Laskowski et al., 2022) and offer insights regarding the behavioural phenotypes and behavioural syndromes most likely to persist under scenarios of increased anthropogenic disturbance (Schell et al., 2021).

Accounting for intraspecific variation has become increasingly important for successful conservation (Des Roches et al., 2021; Merrick & Koprowski, 2017). Although management plans often use ‘average’ (i.e. species level) behaviour and diet data to support decisions, individual variation in Grinnellian and Eltonian factors may be linked to particular phenotypes that disproportionately contribute to a population's dynamics or persistence (Costa‐Pereira, Toscano, et al., 2019; Durell, 2000; Montgomery et al., 2018). Work on the individuality of habitat selection and movement has found that intraspecific variation can be stronger than that of even interspecific differences (Harrison et al., 2019; Montgomery et al., 2018). Indeed, considering animal individuality in conservation efforts is emerging as an effective solution for mitigating human–wildlife conflict, which is often driven by particular phenotypes (e.g. bold individuals) (Barrett et al., 2019; Honda et al., 2018). The inherent link between individual movements and community dynamics holds an increasingly important role in conservation planning and management, thereby potentially advancing our ability to effectively address the global biodiversity crisis (Kays et al., 2015; Merrick & Koprowski, 2017; Nathan et al., 2022).

## 9 CHALLENGES

Multi‐species tracking datasets are becoming more commonplace (Nathan et al., 2022; Wilmers et al., 2015), yet financial, technical and operational challenges must be overcome if quantifying Eltonian and Grinnellian factors from movement data is to become conventional practice. First, these opportunities are currently limited to species capable of carrying tracking devices. New technological developments (e.g. icarus.mpg.de), however, are poised to expand the catalogue of species and guilds possessing movement data (Jetz et al., 2022; animallives.org). Importantly, some ecological actors will inevitably be missed since it is impossible to track every individual in a community, and these missed actors could induce substantial bias in community‐level inference. For example, Creel et al. (2013) found that failing to account for missed interactions between GPS‐collared predators and prey could underestimate antipredator behavioural responses by an order of magnitude or more. This challenge can be further intensified when interacting species exhibit movement patterns across disparate spatial scales (Suraci et al., 2022). For instance, a recent study of wide‐ranging wolves *Canis lupus* and more sedentary elk *Cervus elaphus* recorded more than 36,000 and 13,000 GPS locations of these species, respectively, but documented only 453 ‘encounters’ where conspecifics were co‐located within 1,000 m of each other (Cusack et al., 2020). Addressing these challenges will be system specific and likely involve combining experimental designs that minimize sampling bias with probabilistic modelling (Farage et al., 2021; Gupte et al., 2022; Schlägel et al., 2019), individual‐based simulation (King et al., 2018) and auxiliary data to ‘fill‐in’ critical gaps for unsampled individuals (e.g. camera traps and video‐enabled tracking devices capable of capturing interspecific and intraspecific interactions with unmarked individuals).

Although such auxiliary data provide contextual biological information that compliment movement data, there is still a critical challenge of matching spatiotemporal resolution of multiple data types, which can be quite disparate (e.g. minute‐level resolution of movement data and season‐level resolution of stable isotope data). This challenge is not trivial, but new data fusion algorithms are rapidly developing (Brum‐Bastos et al., 2020; Gupte et al., 2022; Marvin et al., 2016). We expect such methods to constitute an important area of growth moving forward. Furthermore, because modern GPS‐tracking devices can capture data at temporal resolutions of minutes or less, tracking individuals from multiple species over appreciable ecological timescales causes the number of data points soar into the tens of millions. Recent single‐species work on social group dynamics, however, has laid the methodological and analytical foundations from which massive amounts of individual level‐data can be processed, analysed and interpreted (King et al., 2018; Silk et al., 2018; Strandburg‐Peshkin et al., 2015). Thus, the foundation for managing this volume of the data as well as the interpretability and validation of results has been laid and additional, and novel methods that are crucial for operationalizing an individual‐to‐community framework are developing rapidly (Gupte et al., 2022; Nathan et al., 2022; Noonan et al., 2021; Tuia et al., 2022).

The benefit of multispecies tracking datasets appears to be increasingly acknowledged and employed. For example, by tracking several species at the same time in same region, key breeding and foraging sites and migratory routes needed to sustain multiple species at a single site can be identified (Davies et al., 2021; Kauffman et al., 2021; Lowther et al., 2015). However, the tracking of multiple species in the same location is often conducted in isolation by different research groups and the data are typically not well integrated (Nathan et al., 2022). Such scenarios likely stem from differing research agendas and the substantial financial and logistical challenges associated with deploying large numbers of tracking devices on multiple co‐occurring species, thereby highlighting the need for collaboration and data sharing if we are to move towards a more community‐focused use of movement data (Davidson et al., 2020; Kays et al., 2022; Nathan et al., 2022; Urbano et al., 2021). Indeed, coordinating the field logistics alone for multi‐species tracking studies comprises a major challenge that will likely only be overcome through increased coordination among research teams and an inclusive, transparent and collaborative approach to data collection and sharing.

## 10 CONCLUDING REMARKS AND FUTURE VISION

The historical duality between Eltonian and Grinnellian approaches in community ecology has hampered our understanding of how communities are organized in space and time (Soberón, 2007). Additionally, despite growing evidence of the critical role of individual variation in community‐level processes (Bolnick et al., 2011; Costa‐Pereira, Araújo, et al., 2019; Milles et al., 2020), empirical studies are still largely species oriented. These shortcomings stem in part from our inability to simultaneously quantify Eltonian and Grinnellian factors at the individual level in a multi‐species context. We hope the framework presented here will help ecologists overcome these shortcomings by simultaneously capturing interactions between organisms (conspecifics and heterospecifics) and their environment.

This individual‐level framework outlined here should complement—and needs to be complemented by—more traditional population‐ and community‐level approaches to estimate key parameters such as population size, phenotypic trait distribution and local community structure. Combining these data sources certainly entails overcoming major logistical, financial and analytical challenges, yet we are optimistic that recent and future technical developments are beginning to coalesce into Hutchinson's dream toolbox: a toolset capable of simultaneously quantifying multiple niche dimensions across multiple coexisting species. The intuitive, but still largely unexplored, integration between animal movements and community ecology has the potential to shed light on long‐standing questions in ecological theory and help develop new predictive models for gauging the effects of global change on communities.

## CONFLICT OF INTEREST

The authors have no conflict of interest to declare.

## AUTHORS' CONTRIBUTIONS

All authors conceived the ideas, wrote collectively the manuscript and contributed critically to the drafts. We all gave final approval for publication.

## Data Availability

Data will not be archived because this manuscript does not use any data.
